# Subthalamic Single Cell and Oscillatory Neural Dynamics of a Dyskinetic Medicated Patient With Parkinson's Disease

**DOI:** 10.3389/fnins.2020.00391

**Published:** 2020-04-24

**Authors:** Musa Ozturk, Heet Kaku, Joohi Jimenez-Shahed, Ashwin Viswanathan, Sameer A. Sheth, Suneel Kumar, Nuri F. Ince

**Affiliations:** ^1^Department of Biomedical Engineering, University of Houston, Houston, TX, United States; ^2^Department of Neurology, Icahn School of Medicine at Mount Sinai, New York, NY, United States; ^3^Department of Neurosurgery, Baylor College of Medicine, Houston, TX, United States; ^4^Department of Neurology, Baylor College of Medicine, Houston, TX, United States

**Keywords:** Parkinson's disease, subthalamic nucleus, levodopa, dyskinesia, single unit activity, local field potentials

## Abstract

Single cell neuronal activity (SUA) and local field potentials (LFP) in the subthalamic nucleus (STN) of unmedicated Parkinson's disease (PD) patients undergoing deep brain stimulation (DBS) surgery have been well-characterized during microelectrode recordings (MER). However, there is limited knowledge about the changes in the firing patterns and oscillations above and within the territories of STN after the intake of dopaminergic medication. Here, for the first time, we report the STN single cell and oscillatory neural dynamics in a medicated patient with idiopathic PD using intraoperative MER. We recorded LFP and SUA with microelectrodes at various depths during bilateral STN-DBS electrode implantation. We isolated 26 neurons in total and observed that tonic and irregular firing patterns of individual neurons predominated throughout the territories of STN. While burst-type firings have been well-characterized in the dorsal territories of STN in unmedicated patients, interestingly, this activity was not observed in our medicated subject. LFP recordings lacked the excessive beta (8–30 Hz) activity, characteristic of the unmedicated state and signal energy was mainly dominated by slow oscillations below 8 Hz. We observed sharp gamma oscillations between 70 and 90 Hz within and above the STN. Despite the presence of a broadband high frequency activity in 200–400 Hz range, no cross-frequency interaction in the form of phase-amplitude coupling was noted between low and high frequency oscillations of LFPs. While our results are in agreement with the previously reported LFP recordings from the DBS lead in medicated PD patients, the sharp gamma peak present throughout the depth recordings and the lack of bursting firings after levodopa intake have not been reported before. The lack of bursting in SUA, the lack of excessive beta activity and cross frequency coupling between HFOs and lower rhythms further validate the link between bursting firing regime of neurons and pathological oscillatory neural activity in PD-STN. Overall, these observations not only validate the existing literature on the PD electrophysiology in healthy/medicated animal models but also provide insights regarding the underlying electro-pathophysiology of levodopa-induced dyskinesias in PD patients through demonstration of multiscale relationships between single cell firings and field potentials.

## Background

Mapping of the basal ganglia during the implantation of deep brain stimulation (DBS) leads continues to provide researchers insights into the electrophysiology and network dynamics of movement disorders. It is well-known that the structures traversed during intraoperative microelectrode recordings (MER), such as subthalamic nucleus (STN), show distinct patterns both in their spiking (Magariños-Ascone et al., 2000; Rodriguez-Oroz et al., 2001; Starr et al., 2003; Gross et al., 2006; Wong et al., 2009) and oscillatory activity (Wang et al., 2014, 2016; Telkes et al., 2016, 2018) in unmedicated Parkinson's disease (PD) patients. Studies investigating the somatotopic organization of the STN through single cell recordings consistently report that most of the single cell activity related to upper and lower limb movements are located in the dorsolateral region of the STN (Rodriguez-Oroz et al., 2001; Abosch et al., 2002; Theodosopoulos et al., 2003; Weinberger et al., 2006), which consequently have been utilized to target the STN more accurately (Starr et al., 2003). More recent studies have shown that among various spiking profiles that are distributed throughout the STN, bursting and tonic (regular) firings are associated with the symptoms of PD (Sharott et al., 2014; Kaku et al., 2019b). Research in non-human primates also show that the firing type and rate changes in STN neurons can be indicative of healthy, PD or medicated state (Bergman et al., 1994; Soares et al., 2004; Gilmour et al., 2011).

The spatio-spectral characteristics of local field potentials (LFPs) recorded from the same microelectrodes have also been investigated in the territories of STN (Wang et al., 2014; Telkes et al., 2016; van Wijk et al., 2017). Excessive oscillations discovered in the 8–30 Hz (beta) (Priori et al., 2004; Kühn et al., 2006; Chen et al., 2007; Ray et al., 2008; Eusebio and Brown, 2009) and 200–400 Hz (high frequency oscillations, HFO) bands, and the cross-frequency coupling (CFC) between these bands (Foffani et al., 2003; Lopez-Azcarate et al., 2010; Özkurt et al., 2011; Hirschmann et al., 2016; van Wijk et al., 2017; Telkes et al., 2018; Ozturk et al., 2019), have been shown to correlate with the motor symptoms (van Wijk et al., 2016; Shreve et al., 2017; Ozturk et al., 2019) and subtypes of the disease (Telkes et al., 2018).

The effect of dopaminergic medication on LFP patterns of the STN has been studied through the postoperative recordings from chronic DBS leads (macroelectrodes). In particular, the excessive beta peak seen in 8–30 Hz range is suppressed upon dopaminergic medication intake (Brown et al., 2001; Williams et al., 2002; Alonso-Frech et al., 2006; Lopez-Azcarate et al., 2010; Swann et al., 2016; Ozturk et al., 2019). Additionally, the HFO activity seen between 200 and 300 Hz shifts to 300 and 400 Hz frequency range and CFC disappears concurrently (Lopez-Azcarate et al., 2010; van Wijk et al., 2016; Ozturk et al., 2019). LFP peaks in 70–90 Hz range in the STN have also been reported and have been associated with voluntary movement (Cassidy et al., 2002; Cheyne et al., 2008; Thompson et al., 2014) and levodopa-induced dyskinesias (Swann et al., 2016). This modulation of LFPs by PD medication has led to LFP features being used as neuro-biomarkers for closed loop STN stimulation, in which these objective measures facilitate the development of individualized therapies (Hoang et al., 2017; Meidahl et al., 2017; Parastarfeizabadi and Kouzani, 2017).

Although single unit activity (SUA) and LFPs can be recorded simultaneously from microelectrodes, DBS surgeries are typically performed in the unmedicated state. Therefore, the firing profiles of single cells and the spatio-spectral dynamics of microelectrode LFPs *en route* to and in the territories of STN from medicated subjects remain unexplored. Here we report both SUA and LFP characteristics of a PD patient who underwent bilateral STN DBS implantation while “ON” medication. Our observations from this unique case have the potential to fill the gap between studies from healthy or medicated animal models and PD patients, and provide clues regarding electro-pathophysiology of dyskinesias in medicated patients through single cell and field potential modulations.

## Methods

### Case Presentation

A 60 year old, right-handed male with idiopathic PD for 16 years was presented for bilateral STN DBS due to bothersome motor fluctuations and severe generalized choreiform and dystonic dyskinesias despite medication optimization. Pre-operatively, his unmedicated Movement Disorders Society Unified Parkinson's Disease Rating Scale (MDS-UPDRS) PartIII score was 53, which reduced to 12 with medication. Though it is requested that patients withhold their dose of dopaminergic agents 12 h prior to the surgery, the patient took his regular dose of PD medication (1 capsule of Amantadine 100 mg, 3 capsules of Carbidopa and Levodopa extended release 61.25–245 mg) in the morning, 2 h before the surgery. During the recordings, the patient exhibited generalized choreiform dyskinesia. The microelectrode recordings were performed before the placement of the DBS lead, per standard clinical procedure. Starting from left hemisphere, microelectrode recordings were completed in 10 min and DBS lead was placed to test the patient intraoperatively. Same procedure was followed for the right hemisphere 15 min after testing the previous side. Compared to baseline, the patient improved clinically by 51% (pre-op OFF Med: 35, post-op OFF Med/ON Stim: 17) with the electrical stimulation of STN, without any side effects according to the MDS-UPDRS PartIII score (sum of lateral items from both hemispheres: 3.3–3.8, 3.15–3.17) in 3-month follow up. The patient provided informed consent for these recordings under protocols approved by Institutional Review Boards of the University of Houston and Baylor College of Medicine. Additionally, written informed consent was obtained from the individual for the publication of any potentially identifiable images or data included in this article.

### Recordings

The stereotactic coordinates and trajectories to the STN were identified by fusing preoperative magnetic resonance imaging (MRI) and computerized tomography (CT) scans on a neuro-navigational platform (StealthStation, Medtronic, Ireland). This radiographic target was validated using electrophysiological recordings from microelectrodes (NeuroProbe, AlphaOmega, Israel) to identify single unit and LFP characteristics of the STN, per standard clinical protocol. The microelectrodes were initially placed at 15 mm above the target and advanced toward the target in 0.5–1 mm steps using NeuroOmega drive (AlphaOmega, Israel). At each depth, by using the cannula as reference, at least 20 s of SUA from the tungsten tip and LFP from the stainless-steel ring (3 mm above the tip) on the shaft were obtained simultaneously. The dorsal and ventral borders of STN were determined by an experienced neurologist via visual and auditory inspection of SUA activity. The dorsal STN border was identified with a prominent increase in the background activity and this border was used as reference (0 mm). Depths up to 2.5 mm ventral to the dorsal border were referred as the “dorsal” half of STN (Telkes et al., 2018; Kaku et al., 2019b). The SUA recordings were obtained with Grapevine bioamplifier (Ripple, UT) at 30 KHz and LFPs were obtained with the same amplifier at 2 KHz sampling frequency and 16 bit A/D resolution. The data was stored in a computer hard-drive for offline processing.

### Signal Processing

The recorded signals were processed in MATLAB version R2018a (Mathworks, MA). Following the 2nd order IIR high pass filtering of raw SUA data over 300 Hz, spike analyses were performed automatically by the unsupervised pipeline we proposed recently (Kaku et al., 2019a,b) based on two features, namely Local Variation (LV, Shinomoto et al., 2009) and Bursting Index (BI, Cotterill et al., 2016). Note that BI value 0 indicates no burstiness, whereas 1 is the maximum burstiness. Similarly, LV value approaches to 0 for tonic firings whereas 1 refers to the maximum irregularity. Given the number of isolated neurons, each neuron was visualized manually as well, observing and verifying their classification using raw SUA traces as well as inter-spike interval (ISI) histograms. LFP traces were analyzed in the spectral domain using a modified Welch periodogram. A fast Fourier transform was computed at each depth with a 1 s Hamming window and 50% overlap. A median spectrum was calculated from the spectra to eliminate localized artifacts at each depth. Then, individual spectra across depths were combined to generate a 2D depth-frequency map (DFM) representing the depth-varying power spectrum of the LFPs (Telkes et al., 2016). CFC was estimated using a method based on the phase-locking principle (Penny et al., 2008). The comodulograms representing the CFC have amplitude frequency axis from 190 to 410 with 10 Hz steps and 50 Hz filter bandwidth and phase frequency axis from 4 to 40 with 1 Hz steps and 3 Hz filter bandwidth.

## Results

**Figure 1:**
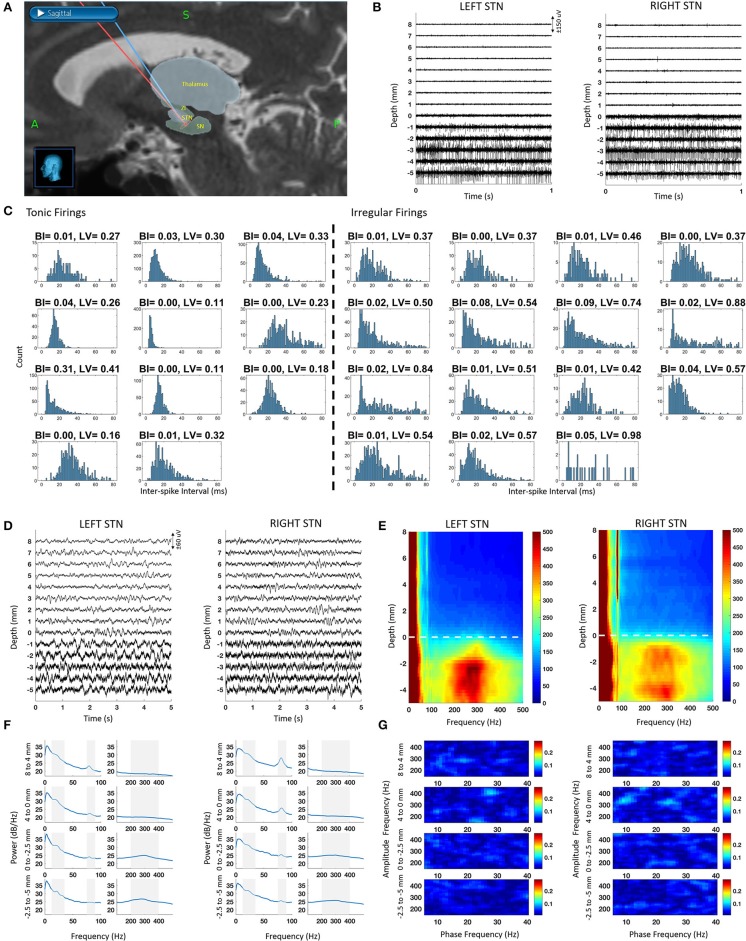
Bilateral SUA and LFP characteristic from intraoperative microelectrode recordings along with the reconstructed trajectories. The depth 0 mm indicate the dorsal border of STN. **(A)** Fused intraoperative MRI and intraoperative CT images used for reconstructed microelectrode trajectories en route to STN in StealthStation, superimposed with relevant structures from the Schaltenbrand-Bailey atlas. **(B)** Sample SUA raw traces with 1 mm step. **(C)** ISI histograms of tonic (regular) and irregular firings as well as their BI and LV values. **(D)** Sample LFP raw traces with 1 mm step. **(E)** DFM showing spatio-spectral profile of LFP recordings. Dashed line represents the dorsal border of STN **(F)** PSD of depth LFPs with 2–4 mm steps. Shaded areas correspond to beta (10–35 Hz), gamma (75–90 Hz), and HFO (200–400 Hz) bands. **(G)** Comodulograms with 2–4 mm steps, representing coupling in the LFP data with 2–4 mm steps.

The raw SUA and LFP data recorded from both hemispheres along the trajectory to the STN are illustrated in Figure 1. Based on the fused intraoperative MRI and intraoperative CT (Figure 1A), the microelectrodes first passed through the anterior thalamus (reticular nucleus) at around 10 mm above dorsal border. Then, the ventral basal complex of the thalamus was encountered, followed by the zona incerta and STN. The lead trajectories were slightly asymmetric due to anatomical and clinical considerations. The SUA raw traces are given in Figure 1B. Similar to the unmedicated recordings, STN was characterized with increased background activity and single unit firings. It was observed that the single neuronal firing and oscillatory activity spatial profiles were similar between hemispheres. Specifically, the prominent increase in the background activity and firing rate in SUA traces were associated with larger signal amplitude in raw LFP traces (Figure 1D).

Nineteen neurons were isolated in the left STN and seven in the right, totaling 26 neurons in both STNs. The ISI histogram as well as BI and LV values for each tonic and irregularly firing neuron is provided in Figure 1C. The mean firing rate of all neurons was 43 ± 15 Hz. Among all isolated neurons, 43% of them demonstrated tonic firing with a mean firing rate of 50 ± 16 Hz whereas 57% fell into an irregular category with a mean firing rate of 38 ± 12 Hz. Spatially, the irregular neurons were distributed equally across the dorsal and ventral STN (50% dorsal and 50% ventral). Tonic firing patterns, however, were mostly found in the ventral STN (11% dorsal and 89% ventral). Based on low BI values (<0.31) and the visual observation of the ISI histograms and the raw traces, no burst-type firings were observed.

The raw traces, depth-frequency maps and the power spectra of LFP at various depths are provided in Figures 1D–F. Throughout the recordings, LFP activity included a dominant low frequency component (<8 Hz) and a sharp gamma (70–90 Hz) activity. Interestingly, the power of the gamma band was stronger out of STN extending >10 mm above it, especially on the right hemisphere, likely due to the larger overlap with the thalamic region on this side (Figure 1A). In both left and right hemisphere spectra, the gamma peak was consistent. Above the STN, this narrow peak was significantly higher than the median power in band with 20 Hz surrounding it (Wilcoxon rank sum test, *p* < 0.001). In-STN, the peak was distinguishable but not significantly different than the median (Wilcoxon rank sum test, *p* > 0.05). We also observed another peak with a broad band characteristic between 200 and 400 Hz (HFO) in the STN. The peak of this band was significantly higher than the median power of the band with 200 Hz surrounding it (Wilcoxon rank sum test, *p* < 0.05). The peak in the beta range was also visible but not significantly elevated with respect to its surroundings (Wilcoxon rank sum test, *p* > 0.05). Despite the presence of strong oscillatory activity in the high frequency range (200–400 Hz), no cross-frequency coupling in the form of phase-amplitude modulation between beta and HFO bands was detected (Figure 1G).

Following MER, the STN DBS lead was placed and intraoperative test stimulation was performed at constant frequency and pulse width (130 Hz, 60 μs) with voltages from 1 to 5 V. No side effects were encountered but contralateral dyskinesia improved (Supplementary Video available for the testing of left hemisphere) and recurred with cessation of stimulation.

## Discussion

In this case report, we present unique intraoperative depth recordings of single unit firings and neural oscillations from a PD patient who took his full dose of regular medication before the surgery. The patient was a 60 year old male diagnosed with idiopathic PD and was referred to bilateral STN-DBS partly due to severe dyskinesias even after optimized medication treatment. The microelectrode recordings starting from 15 mm above the STN revealed interesting relationships between the SUA and LFP profiles of our medicated patient and healthy/medicated non-human primates and medicated patients.

### No Burst Type Firings in the Medicated State

The identification of different firing types in the STN have been studied previously by others (Bergman et al., 1994; Magariños-Ascone et al., 2000; Soares et al., 2004; Weinberger et al., 2006, 2008; Du et al., 2018). We recently contributed to this field with a clustering method to identify bursting, tonic and irregular firing types of STN neurons in an unsupervised fashion (Kaku et al., 2019b). After recording 279 neurons from 20 STNs of 12 unmedicated patients, we reported that the irregular firings dominate the STN firing profile (55%), followed by bursting (34%) and then tonic (11%) firing regimes (Kaku et al., 2019b). These findings on the proportion of bursting firings were also in accordance with studies involving 1-methyl-4-phenyl-1,2,3,6-tetrahydropyridine (MPTP) treated non-human primates that report an increase in bursting neuronal activity and instantaneous firing rates after MPTP treatment (Bergman et al., 1994; Soares et al., 2004). Strikingly, in our medicated patient, no burst type firing patterns were observed. Instead, tonic firings increased in proportion without losing their spatial distribution. Out of 26 neurons isolated, 57% had irregular firing patterns, and 43% showed tonic firing patterns. The overall mean firing rate (43 ± 15 Hz) was comparable to our recent report on 12 unmedicated patients (47 ± 31 Hz) (Kaku et al., 2019b). Yet, when we compared the firing rate of patterns separately, we found that while the rate of irregular firings was similar (medicated 38 ± 12 Hz vs. unmedicated 34 ± 17 Hz), the rate of tonic firings was clearly slower in our medicated patient (medicated: 50 ± 16 Hz vs. unmedicated: 74 ± 33 Hz). On the other hand, reports on man and non-human primates medicated with apomorphine or levodopa show that population firing rates in the STN did not change with drug intake by itself (Lozano et al., 2000; Levy et al., 2001; Gilmour et al., 2011) but did decrease when dyskinesia was present (Mitchell et al., 1992; Lozano et al., 2000), as it did for our medicated patient. Therefore, our observations regarding the firing rate changes could be attributed to not only the therapeutic effect of the drug (MDS-UPDRS part III OFF-ON: 53-12) but also the dyskinetic state it induced. It should be noted that a study involving dyskinetic patients treated with apomorphine reported an *increase* in “burstiness” in single cell recordings (Levy et al., 2001) which contradicts with our finding of no bursting. They, however, noted that the increase was due to aperiodic bursting. Regardless, this difference could perhaps be due to how levodopa and apomorphine differ in their mechanisms of action (Gilmour et al., 2011). Future studies with more subjects exploring firing patterns before and during dyskinesia periods in subjects administered with levodopa or apomorphine will be able to reach to a more definitive conclusion.

### Oscillatory Neural Dynamics in the STN

Studies investigating STN-LFP oscillations from the DBS lead in medicated patients (Brown et al., 2001; Williams et al., 2002; Alonso-Frech et al., 2006; Lopez-Azcarate et al., 2010; Özkurt et al., 2011; Swann et al., 2016; Ozturk et al., 2019) have shown that dopaminergic medication suppresses the prominent beta peak and 200–300 Hz HFO in the STN while new peaks may appear in 70–90 Hz gamma and 300–400 Hz HFO range, similar to MPTP treated healthy non-human primates (Escobar et al., 2017). In line with these reports, we observed weak beta activity, a sharp gamma peak at 80 Hz and a strong broadband HFO centered around 300 Hz in STN, as shown in Figures 1E,F. Furthermore, we and others have shown that there is coupling between beta and 200–300 Hz HFO bands in the unmedicated patients that disappears with dopaminergic medication intake (Lopez-Azcarate et al., 2010; Özkurt et al., 2011; van Wijk et al., 2016; Ozturk et al., 2019). Similarly, this coupling was absent in our medicated patient as well. Lack of bursting firings in the SUA and the absence of CFC in the LFP subbands could be an indication of a multiscale relationship between single neuronal and oscillatory activity in PD (Yang et al., 2014; Meidahl et al., 2019).

### The Role of Finely Tuned Gamma

The 70–90 Hz sharp gamma activity in the STN and in the motor cortex has been labeled as “prokinetic” due to its enhancement with voluntary movement (Cassidy et al., 2002; Cheyne et al., 2008; Thompson et al., 2014). Studies recording together from cortex, pallidum, and STN have also found coherence at the sharp gamma activity between these sites (Cassidy et al., 2002; Williams et al., 2002; Kempf et al., 2009). Unlike previous studies from chronic DBS lead or cortical electrodes, microelectrode recordings shown here sample from various depths above and within STN. Note that in Figures 1E,F, the sharp gamma is not only present within the STN but even up to 10 mm above the dorsal border, overlapping with thalamus, indicating a broader network synchronization. Furthermore, the sharp gamma activity appears more peaked above the STN in both hemispheres. A previous study recording the same activity from STN and cortex simultaneously reported that it was stronger in cortex (Swann et al., 2016). Another study recording from thalamus and pallidum observed stronger sharp gamma in the thalamus (Kempf et al., 2009). Similarly, the difference in power of the gamma band in our recordings could be stemming from the structures it is recorded from, as we traversed anterior thalamus, zona incerta and arrived at STN. Combined with the cortical recordings, we can speculate that the sharp gamma activity could be present with varying power levels from cortex to subcortical areas. Thus, with its recurring presence in the subcortico-cortical loop, the finely tuned gamma activity might be serving as an “*assembling rhythm”* between the structures (Buzsáki et al., 2012) and providing a communication channel between them (Fries, 2015). Alternatively, combined with dominant slow oscillations below 8 Hz (Alonso-Frech et al., 2006), the exaggerated gamma activity might also be contributing to the levodopa-induced dyskinesia (LID) generation (Swann et al., 2016). The link between sharp gamma and LID have also been explored at several levels including cortex, striatum and STN of animal models (Halje et al., 2012; Delaville et al., 2015; Belić et al., 2016). Supporting this claim, the patient presented here demonstrated generalized choreiform dyskinesias (Supplementary Video) after levodopa intake which disappeared with STN-DBS (Oyama et al., 2012). It is possible that the -otherwise physiological—gamma rhythm becomes pathological when it is exaggerated due to disrupted “filtering” in the dopamine-overdosed basal ganglia (Weinberger and Dostrovsky, 2011; Swann et al., 2016). Reports of successful thalamotomy/pallidotomy procedures on patients with LID (Narabayashi et al., 1984; Jankovic et al., 1999; Guridi et al., 2012) also support that a circuit level mechanisms is involved in the generation of dyskinesia (Swann et al., 2016; Cenci et al., 2018). Paired with the appropriate processing techniques, sharp gamma oscillations can be used to develop closed-loop technologies to control dyskinesia (Swann et al., 2016).

## Concluding Remarks

Investigating bilateral microelectrode recordings from above and within STN of a medicated PD patient with dyskinesia for the first time, we have observed that there were no burst type firings unlike unmedicated subjects. Instead, tonic firings with rates slower than unmedicated patients increased in proportion. The oscillatory LFP patterns confirmed previous reports from chronic macroelectrodes in medicated patients as the exaggerated beta and beta-HFO coupling was absent despite the presence of strong HFO band activity. Additionally, sharply tuned gamma oscillations in STN were in accordance with reports from medicated or dyskinetic patients but extension of these oscillations out of STN was not reported before either. Together, these preliminary findings help validate the existing healthy/medicated non-human primate literature on the PD electrophysiology in human subjects and provide insights into the electro-pathophysiology of dyskinesia in the medicated PD patients through increased tonic firing profiles and sharp gamma and HFO activity. Despite lack of concrete evidence, the increase in the proportion of tonic firings in STN and concomitant broadband HFO and sharp gamma oscillations after the medication intake implies that these patterns might be related. Simultaneous recordings from out- and in-STN as well as the cortex in future studies with greater number of subjects might unravel the underlying relationship between tonic firings and these LFP bands in the pathophysiology of PD more clearly.

## Data Availability Statement

The data that support the findings of this study are available on a reasonable request from the corresponding author. The raw data are not publicly available as the data might contain potentially identifying or sensitive information that could compromise the privacy of the research participants.

## Ethics Statement

The studies involving human participants were reviewed and approved by University of Houston Baylor College of Medicine. The patients/participants provided their written informed consent to participate in this study. Written informed consent was obtained from the individual(s) for the publication of any potentially identifiable images or data included in this article.

## Author Contributions

NI and JJ-S conceived and designed the experiments. MO, HK, and AV performed the experiments. JJ-S and SK performed clinical testing. MO, HK, and NI analyzed the data. MO and NI wrote the paper. MO, HK, JJ-S, AV, SS, SK, and NI reviewed and revised the manuscript and approved the final manuscript as submitted.

## Conflict of Interest

The authors declare that the research was conducted in the absence of any commercial or financial relationships that could be construed as a potential conflict of interest.

## Supplementary Material

Supplementary Video The intraoperative clinical testing for the left STN at constant frequency and pulse width (130Hz, 60 μs) with voltages from 1 to 5 V. The medicated patient demonstrated generalized choreiform dyskinesias which disappeared after STN-DBS was turned ON. No side effects were observed.
